# Supplementary material for: Arctic sea ice is an important temporal sink and means of transport for microplastic
Source: Nat Commun. 2018 Apr 24;9:1505. doi: 10.1038/s41467-018-03825-5 (PMC5915590; doi:10.1038/s41467-018-03825-5)
Supplement: Supplementary file 1 — Supplementary Information [file 41467_2018_3825_MOESM1_ESM.docx]

**Supplementary Information to:**

**Arctic sea ice is an important temporal sink and means of transport for microplastic**

Peeken et al. 2018

*Corresponding author: ilka.peeken@awi.de

**Supplementary Figures:**

Supplementary Figure 1: Box- and whiskers plots of proportions (%) of synthetic polymers in all ice cores. The boundary of the box closest to zero indicates the 25^th^ percentile, a line within the box marks the median, and the boundary of the box farthest from zero indicates the 75^th^ percentile. Whiskers (error bars) above and below the box indicate the 90^th^ and 10^th^ percentiles. Blue lines indicate the mean and black bullets outliers. Polymer abbreviations: see Supplementary Table 1


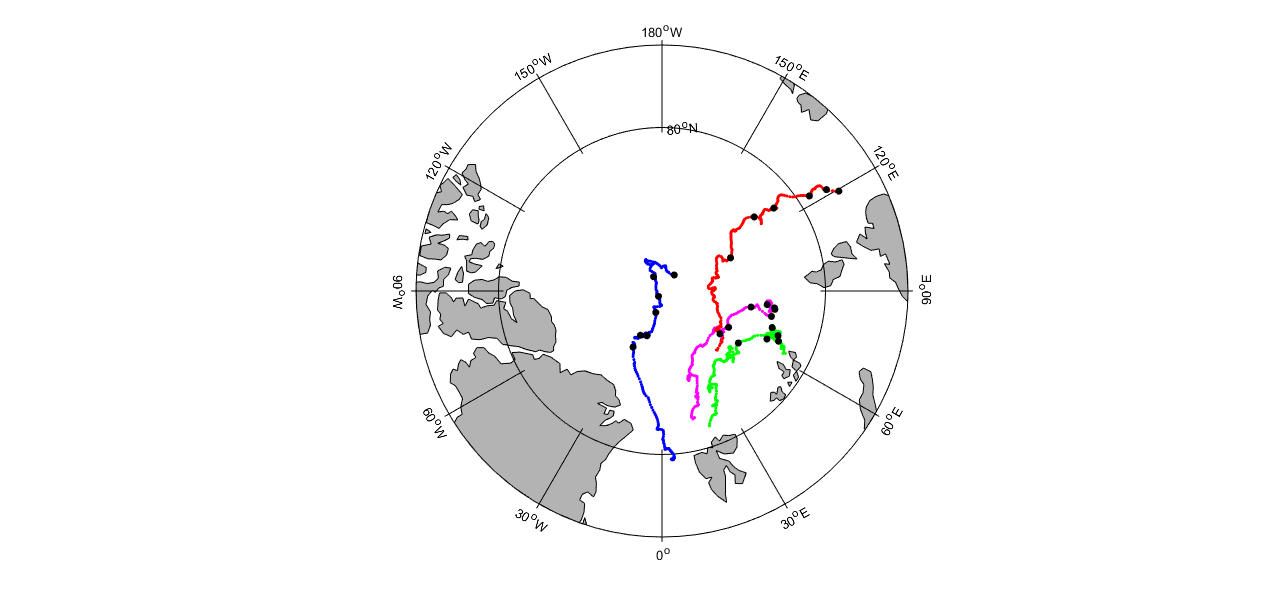


Supplementary Figure 2: Back trajectories of the modelled ice cores as shown in Fig. 1d where black dots represent the mean locations where the ice of the respective vertically resolved samples was formed.


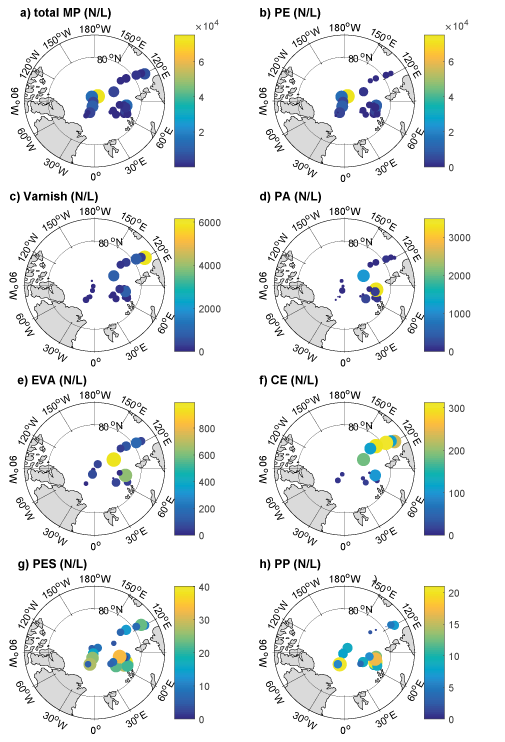


Supplementary Figure 3: (a) Derived regional distribution of total microplastic (MP) concentration given in particles xL^-1^ in all georeferenced ice samples. (b-h) shows the same, but for the different MP species (abbreviations s. Supplementary Table 1; CE refers to cellulose acetate). Colors indicate MP concentration on a linear color bar, while circle diameters are proportional to MP concentration and for a better visibility depicted on a logarithmic scale

**
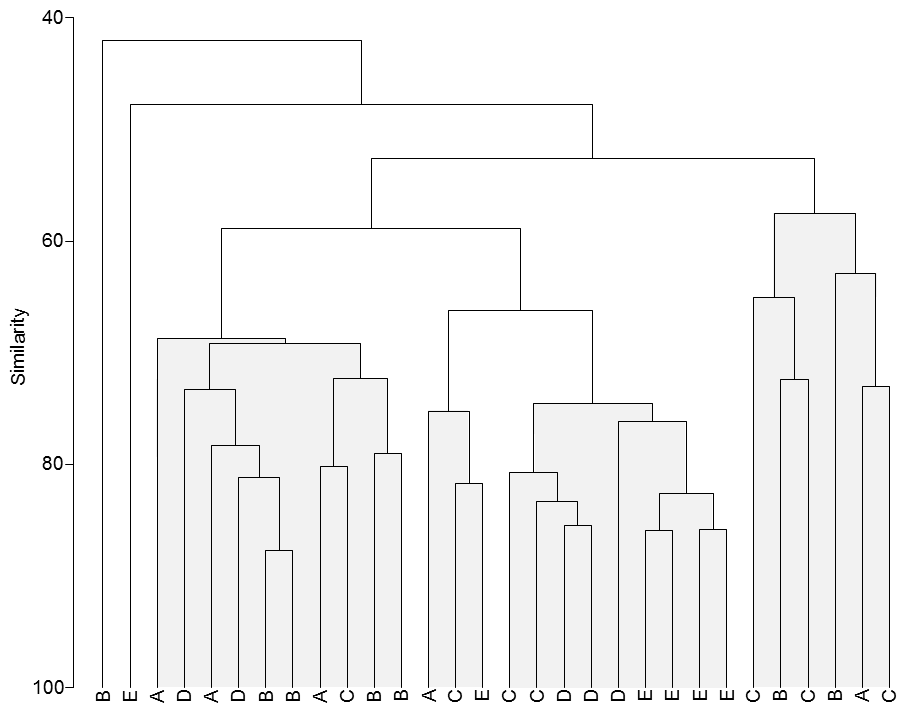
**

Supplementary Figure 4: Cluster analysis (group average) and similarity profiles test (SIMPROF) of polymer specific MP numbers in different sections of ice cores (based on Bray-Curtis similarities). A significance level of 5% was used to test the SIMPROF statistic. Clusters which cannot significantly differentiated by SIMPROF are shaded in grey.

**
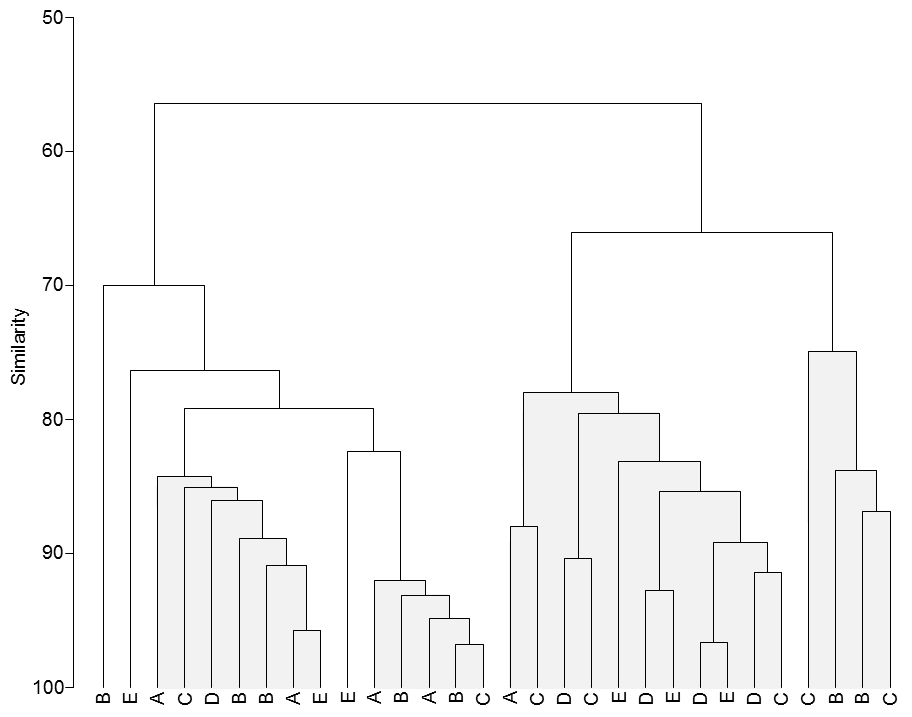
**

Supplementary Figure 5: Cluster analysis (group average) and similarity profiles test (SIMPROF) of MP particles in different size classes in different sections of ice cores (based on Bray-Curtis similarities). A significance level of 5% was applied to test the SIMPROF statistic. Clusters which cannot significantly differentiated by SIMPROF are shaded in grey.

**
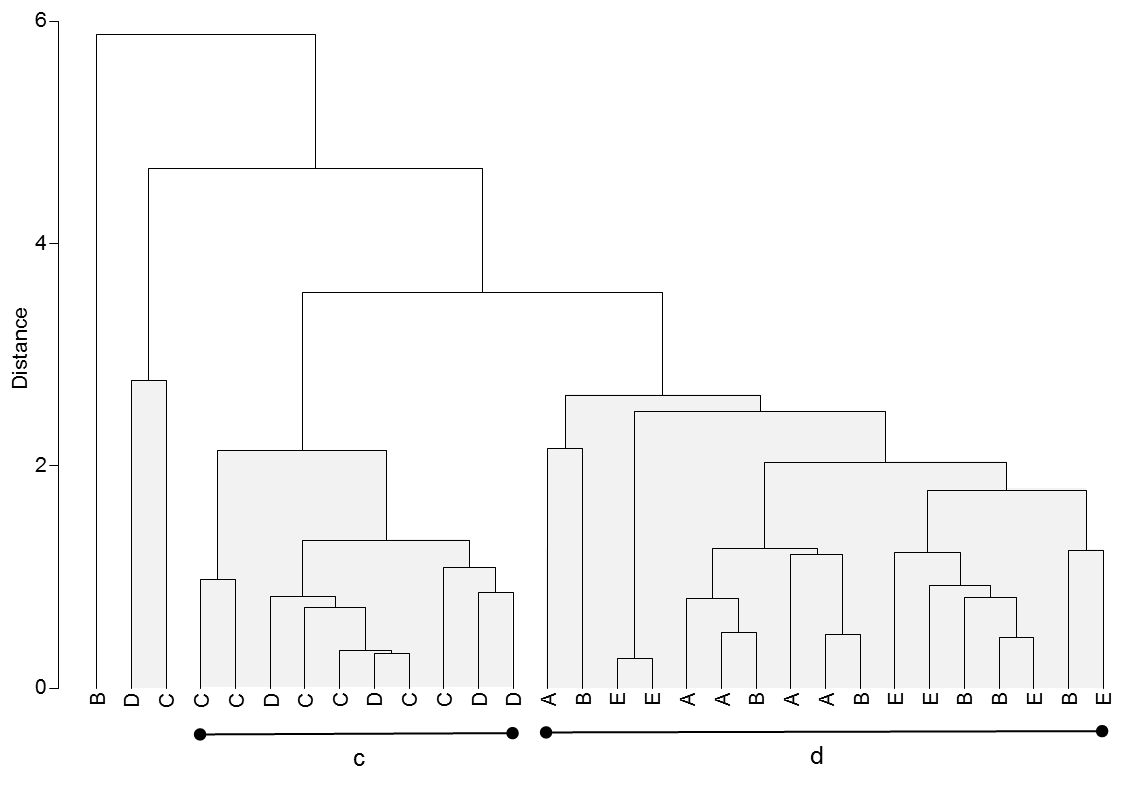
**

Supplementary Figure 6: Cluster analysis (group average) and similarity profiles test (SIMPROF) of ancillary variables in different sections of ice cores (based on Euclidean distances). SIMPROF grouping of samples is indicated by characters “c” and “d*”.* A significance level of 5% was applied to test the SIMPROF statistic. Clusters which cannot significantly differentiated by SIMPROF are shaded in grey.

**Supplementary Tables:**

Supplementary Table 1: Observed polymer distribution and abbreviation of all found polymers. The polymers are ranked according to their percentage of occurrence in all sections of the ice cores (see Supplementary Fig. 1). All polymers below 1% (starting with nitrile rubber) were classed as others in Fig 1c and 5.

Supplementary Table 2: Spearman rank correlations between MP related data (total MP numbers, diversity indices (S: richness; H´: Shannon Wiener-diversity), polymer specific numbers, numbers of particles in size classes) with ancillary variables recorded in different horizons of the ice cores. Significant correlations (p<0.05) are highlighted in bold. (Chlorophyll a = Chl a [µg L^-1^], Particulate organic nitrogen = PON [mg L^-1^], Particulate organic carbon = POC [mg L^-1^], carbon/nitrogen ratio =C/N ratio [weight/weight], Temperature [°C], Salinity [PSU.

Supplementary Table 3: Results of ANOVA tests of ancillary variables. Displayed are tests for the factor SIMPROF^av^. P-values were obtained using type III sums of squares; significant results (p < 0.05) are highlighted in **bold**. SS: sums of squares; MS: mean square; Chla: Chlorophyll a; PON: Particulate organic nitrogen; POC: Particulate organic carbon; C/N: carbon/nitrogen ratio
