# Supplementary material for: Arctic sea ice is an important temporal sink and means of transport for microplastic
Source: Nat Commun. 2018 Apr 24;9:1505. doi: 10.1038/s41467-018-03825-5 (PMC5915590; doi:10.1038/s41467-018-03825-5)
Supplement: Supplementary file 3 — Description of Additional Supplementary Files [file 41467_2018_3825_MOESM3_ESM.pdf]

Description of Additional Supplementary Files

File Name: Supplementary Data 1
Description: ANOVA main tests of total MP numbers, richness (S), Shannon Wiener-diversity (H´) and polymer specific particle numbers. Displayed are tests for the factor SIMPROFav. p-values were obtained using type III sums of squares; significant results ($p < 0.05$) are highlighted in bold. SS: Sums of squares; MS: Mean square

File Name: Supplementary Data 2
Description: ANOVA main tests of MP numbers in different size classes. Displayed are tests for the factor SIMPROFav. p-values were obtained using type III sums of squares; significant results ($p < 0.05$) are highlighted in bold. SS: Sums of squares; MS: Mean square
